# Underfed Children

**Published:** 1906-02-24

**Authors:** 


### UNDERFED CHILDREN.

UNLESS the Relief (School Children) Order is much amended it will certainly be allowed to lapse into desuetude. So far it has resulted in a great deal of work, a very limited sum of good, and a considerable amount of confusion. The order having arranged for the feeding of children at school, no provision is made for holidays. If the children cannot obtain food at home during school time, there is no reason to expect that the condition of the family exchequer will improve when they are at home. Yet for this no provision is made by the order. Even if the order allowed for children being fed in holiday time, it is not to be expected that teachers would give up their leisure to see to the feeding of the children—a task which is labelled "voluntary," but would be more accurately entitled simply "unpaid." Nor can it be said that even where it is operative the order has been satisfactory in its results. In the Wandsworth Union the clerk told the Guardians that a hundred cases had been reported and inquired into by the relieving officers in the different districts. From these inquiries it appeared:—(*a*) That several of the cases were unknown at the address given; (*b*) That only in one or two cases was there anything approaching underfeeding; (*c*) That in many cases the parents resented the relieving officer calling upon them; (*d*) That in the majority of cases there was nothing to warrant the names being put down as underfed; (*e*) That very little inquiry was made by the teachers before putting them on the list. So much for a metropolitan district which, in Wandsworth itself and in Battersea, includes a large number of poor. The same tale comes from the provinces. At Southampton a list of between two and three thousand names had been sent to the relieving officer, who at once said that he could not make proper inquiries unless other officers were appointed to help him. It appeared, however, that meals had been given indiscriminately by a "Needy Children" sub-committee, who as a result of their improvidence were now compelled to suspend the work for lack of funds, and appealed to the Guardians for a grant in aid. This was granted after some discussion, but with a warning that more discretion should be exercised in the distribution of the meals. It certainly seems that far more care is needed than has hitherto been shown if the order is not to degenerate into a means of allowing the selfish and lazy to transfer their responsibilities to the honest and hardworking members of the community.

